# Muscular glucose assessed by microdialysis and blood glucose can predict mortality in septic shock

**DOI:** 10.1186/cc12178

**Published:** 2013-03-19

**Authors:** A Massoudi, M Belhadj Amor, C Romdhani, A Ben Gabsia, I Labbene, M Ferjani

**Affiliations:** 1 Military Hospital of Tunis, Tunisia

## Introduction

The aim of our study was to assess the muscular glucose by microdialysis and its association with mortality in septic shock patients.

## Methods

We conducted a preliminary prospective study. We included septic shock patients hemodynamically optimized according to international recommendations. A microdialysis catheter was inserted in the femoral quadriceps. Interstitial fluid samples were collected every 6 hours for 5 days. The determination of muscular glucose was performed by the CMA 600 analyzer (CMA/Microdialysis AB, Sweden). We also performed a dosage of concomitant blood glucose. The study population was divided into two groups according to hospital mortality. Statistic analysis: Mann-Whitney test and chi-squared test: comparisons between groups. Quantitative variables were expressed as mean ± standard deviation or median (interquartile range) as appropriate.

## Results

We included 12 patients with septic shock. The mortality rate was 50%. Demographics were comparable between groups except for age (66 ± 9 vs. 41 ± 12, dead patients vs. survivors, respectively; *P *= 0.002). Pneumonia was the major cause of septic shock (10 patients). We analysed 167 blood samples and 166 muscular glucose samples. We found a positive association between muscular glucose, blood glucose and mortality. Tissue glucose was significantly higher among dead patients compared with survivors at the 54th hour. Comparing all data, muscular glucose (*P *= 0.02) and blood glucose (*P *= 0.007) were significantly higher in dead patients (Table 1).

**Table 1:** Association between muscular glucose, blood glucose and mortality

	Dead patients (*n *= 6)	Survivors (*n *= 6)	*P *value
Muscular glucose (mmol/l)	9.4 (5.66; 13.71)	7.87 (5.62; 10.41)	0.02
Blood glucose (mmol/l)	10.9 (8.3; 15.5)	8.6 (6.9; 11.4)	0.007

## Conclusion

Our data suggest that muscular glucose assessed by microdialysis and blood glucose are associated with mortality in septic shock patients. Therefore, muscular glucose may reflect the metabolic alterations and microcirculatory dysfunction induced by septic shock.

